# Diagnostic Yield of Fecal Immunochemical Test for Advanced Colorectal Neoplasms in Adults Under 50: A Single-Center Cohort in Taiwan

**DOI:** 10.3390/jcm15114293

**Published:** 2026-06-02

**Authors:** Chi-Chu Lo, Yen-Ling Chiu, Pao-Shu Wu, Ai-Hsien Li, Chen-Huan Yu, Cheng-Lu Lin, Chen-Shuan Chung, Chien-Chu Lin, Kuan-Chih Chen

**Affiliations:** 1 Division of Gastroenterology and Hepatology, Department of Internal Medicine, Far Eastern Memorial Hospital, New Taipei City 220, Taiwan; femh97061@femh.org.tw (C.-C.L.); femh96646@femh.org.tw (C.-H.Y.); lamusa1983@gmail.com (C.-L.L.); chungchenshuan_3@yahoo.com.tw (C.-S.C.); s0316@ms10.hinet.net (C.-C.L.); 2 Health Management Center, Far Eastern Memorial Hospital, New Taipei City 220, Taiwan; yenling.chiu@gmail.com (Y.-L.C.); las1012.tw@gmail.com (A.-H.L.); 3 Graduate Institute of Medicine, Yuan Ze University, Taoyuan 320, Taiwan; 4 Department of Anatomic Pathology, Far Eastern Memorial Hospital, New Taipei City 220, Taiwan; pw2136@gmail.com; 5 School of Nursing, Yuan Ze University, Taoyuan 320, Taiwan; 6 Program A, Department of Electrical Engineering, Yuan Ze University, Taoyuan 320, Taiwan; 7 Graduate Institute of Biomedical Electronics and Bioinformatics, National Taiwan University, Taipei 106, Taiwan

**Keywords:** colorectal neoplasms, colorectal adenoma, early detection of cancer, occult blood, young adult

## Abstract

**Background/Objectives**: The incidence of early-onset colorectal cancer is increasing worldwide. The fecal immunochemical test (FIT) is widely used for screening adults aged 50 and older, but its performance in younger individuals is less understood. **Methods**: We retrospectively analyzed 202,676 FITs from asymptomatic adults aged 18–49 between 2011 and 2025. FIT results, age categories, and follow-up colonoscopy findings were evaluated. **Results**: The FIT positivity rate was 4.7%. Among 1973 FIT-positive individuals who underwent colonoscopy, 5.9% had advanced adenoma or sessile serrated lesion and 1.3% had invasive cancer. A total of 143 advanced neoplasms (ANs) were detected, with prevalence increasing with age. Most ANs (79.1%) occurred in those aged 40–49. The prevalence of ANs was higher in the 45–49 than in the 40–44 age group (49.0% vs. 30.1%; OR 1.55, 95% CI 1.04–2.32) and higher in the 40–44 than in the 35–39 age group (30.1% vs. 10.5%; OR 2.20, 95% CI 1.20–4.02). **Conclusions**: The diagnostic performance of FIT in individuals under 50 years is comparable to that observed in the older population. Given the age-related rise in the prevalence of ANs in young adults, several countries have lowered the screening age to 45 years, and extending screening to individuals aged 40 years may be warranted.

## 1. Introduction

Globally, colorectal cancer (CRC) remains a major public health challenge, ranking as the third most frequent malignancy and the second leading cause of cancer-related mortality [1]. According to GLOBOCAN 2022 estimates—incorporating data from Africa, Latin America and the Caribbean, Northern America, Europe, Oceania, and Asia—the global age-standardized incidence and mortality rates for CRC based on the 1966 Segi–Doll World standard population were 18.4 and 8.1 per 100,000, respectively. The disease also accounts for a substantial survival burden, with a 5-year prevalence proportion of 73.2 per 100,000 individuals, representing over 5.7 million people living with the disease worldwide [2]. According to the 2023 Taiwan Cancer Registry data from the Health Promotion Administration, the age-standardized incidence rate for CRC was 43.7 per 100,000. In contrast, the mortality rate—encompassing both colorectal and anal cancers—stood at 14.0 per 100,000; both metrics were adjusted to the 2000 World Health Organization world standard population [3]. Fortunately, the long-term increasing trends in CRC incidence and mortality in the overall population of Taiwan have recently plateaued or begun to show signs of slowing growth, and this trend is mainly attributed to CRC screening in individuals aged 50 years and older during the past two decades [3]. In contrast, the incidence of early-onset CRC (EOCRC), defined as CRC diagnosed in individuals younger than 50 years, continues to rise and warrants close attention worldwide [3,4,5]. EOCRC was ranked as the fourth leading cause of cancer death among both men and women in the late 1990s, and has now risen to the top position in men and second in women [4].

A comprehensive global analysis of CRC incidence in 36 countries revealed that the incidence of EOCRC has increased in 19 countries since 2012; among these countries, nine have declining or stable trends in those aged 50 years or older [6]. For example, in the United States, both the incidence and mortality rates of CRC have declined among individuals aged 50 years and older, in contrast to the increasing trend observed in those aged less than 50 years. Since then, the United States has recommended lowering the screening age to 45 years in 2018 in response to this trajectory [5,7].

Due to the global pattern of increasing EOCRC incidence, the participation of younger adults in CRC screening has become a focus of attention. Worldwide, several options are available for CRC screening, including guaiac fecal occult blood test, fecal immunochemical test (FIT), stool deoxyribonucleic acid test, flexible sigmoidoscopy, colonoscopy, and computed tomography colonography. While the effectiveness and supporting evidence vary for each approach, all CRC screening methods aim to prevent CRC by facilitating the detection and subsequent removal of precancerous lesions [8].

In major countries such as the United States, Europe, parts of the United Kingdom, Australia, and Korea, screenings for the early detection of CRC began at age 50 from the late 1990s to the early 2000s [9]. In Taiwan, the evolution of the biennial FIT-based CRC screening program started in 2004 and also targeted those aged above 50, with considerable success in reducing CRC-related death over the years [10,11]. However, an age-related acceleration in EOCRC was still observed in Taiwan, with the age-specific incidence rate reaching 33.82 per 100,000 population among individuals aged 45–49 years [3]. Notably, according to Chiu et al., initiating CRC screening in individuals aged 40–49 years may provide a more substantial reduction in long-term CRC incidence and mortality compared to the traditional starting age of 50 [12]. Some countries, including the United States, Australia, and Taiwan, have lowered the CRC screening age to 45 years, whereas most countries still commence screening at the age of 50 years [13,14]. In this study, we aimed to evaluate the FIT positivity rate among asymptomatic young adults under 50 years of age and to assess the prevalence of colonic advanced neoplasms (ANs) across different age groups to provide empirical evidence for the necessity of adjusting the screening age.

## 2. Materials and Methods

### 2.1. Participant Recruitment and Study Design

We retrospectively enrolled asymptomatic patients aged between 18 and 49 years who underwent FIT at the Health Management Center of Far Eastern Memorial Hospital between 2011 and 2025. The primary outcome of this study was the FIT positivity rate in young adults, while the secondary outcome focused on the results of subsequent colonoscopies.

### 2.2. FIT Screening Tool

From January 2011 to June 2021, the quantitative FIT kit used was HM-Jack (Kyowa Medex Co., Ltd., Tokyo, Japan). Beginning in July 2021 and continuing through May 2025, it was replaced by an OC sensor (Eiken Chemical Co., Tokyo, Japan). Both kits use a positive cutoff value of 20 μg hemoglobin per gram of feces.

### 2.3. Definitions of Outcomes

Colonic polyps were categorized as neoplastic colonic polyps, hyperplastic polyps, hamartomatous polyps, and inflammatory polyps. Neoplastic colonic polyps were defined as tubular adenoma (TA), tubulovillous adenoma (TVA), villous adenoma, sessile serrated lesion (SSL), or traditional serrated adenoma (TSA) [15]. Advanced adenoma (AA) included adenomas with a size larger than 10 mm, exhibiting tubulovillous or villous architecture, high-grade dysplasia, or intramucosal adenocarcinoma. An advanced sessile serrated lesion (ASSL) was an SSL of 10 mm or larger or with dysplasia [16]. ANs included AA, ASSLs, and invasive cancer [17]. The left side of the colon was defined as the anatomic location in the descending colon, sigmoid colon, and rectum, whereas the remaining part of the colon was classified as the right side of the colon. Positive predictive value (PPV) was calculated as the proportion of individuals with a positive FIT result who were confirmed to have colorectal neoplasia on follow-up colonoscopy. Because not all FIT-positive individuals underwent colonoscopic verification, the PPV reported in this study should be interpreted as a verified PPV, calculated only among those who completed diagnostic colonoscopy.

### 2.4. Statistical Considerations

Discrete data are presented as numbers and percentages. Binary logistic regression was performed using SPSS for Windows (version 30.0; IBM Corp., Armonk, NY, USA) to assess the associations of gender and age categories with FIT results, and the association between age groups and the prevalence of ANs, as well as the impact of time interval between FIT and colonoscopy on the likelihood of invasive cancer detection. The results are presented as odds ratios (ORs) with 95% confidence intervals (CIs). To further evaluate the diagnostic performance of FIT, receiver operating characteristic (ROC) curves were constructed, and the area under the curve (AUC) was calculated. The optimal cutoff values for FIT concentrations were determined using the Youden index. A two-tailed *p* value < 0.05 was considered statistically significant.

## 3. Results

**Figure 1:**
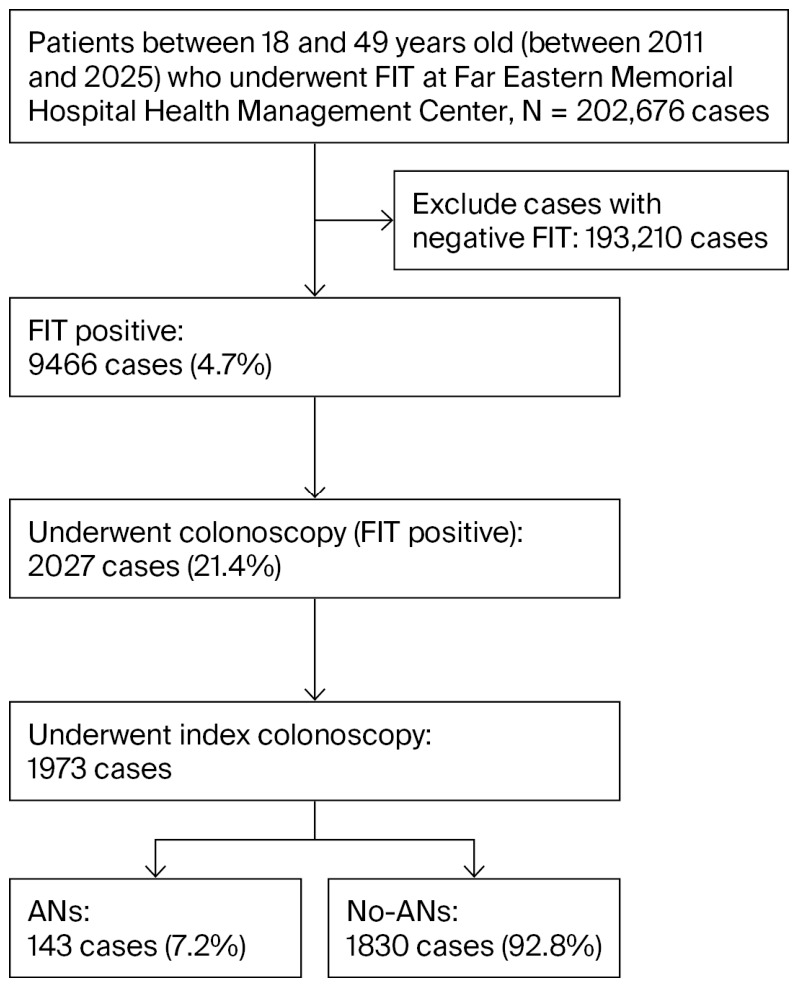
Flow diagram of the study population.

Between January 2011 and May 2025, a total of 202,676 FITs were performed, with 9466 FITs showing positive results. A total of 2027 patients with positive FIT results underwent colonoscopy. After excluding post-index colonoscopy, 1973 individuals remained, 899 of whom (45.6%) were female (Figure 1).

### 3.1. FIT Positivity Rate in Younger Population (Table S1)

**Figure 2:**
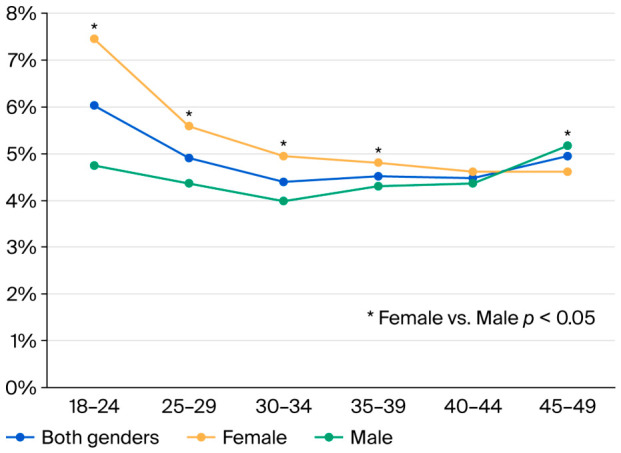
Trend in FIT positivity rate (%) of each age group.

The overall FIT positivity rate was 4.7%, with a slightly higher rate in females than in males (4.94% vs. 4.47%). Across age groups, the positivity rate decreased from 6.03% in the 18–24 age group to 4.91% in the 25–29 age group, and further to 4.40% in the 30–34 age group. It then slightly rebounded in the 35–39 age group (4.52%), declined again in the 40–44 age group (4.48%), and rose once again in the 45–49 age group (4.95%). When stratified by sex, females had significantly higher FIT positivity rates than males did in the 18–24 age group (OR 1.61; 95% CI, 1.29–2.01; *p* < 0.001), 25–29 age group (OR 1.29; 95% CI, 1.16–1.45; *p* < 0.001), 30–34 age group (OR 1.26; 95% CI, 1.14–1.39; *p* < 0.001), and 35–39 age group (OR 1.12; 95% CI, 1.02–1.23; *p* = 0.015). No significant sex difference was detected in the 40–44 age group (OR 1.06; 95% CI, 0.97–1.15; *p* = 0.194), whereas in the 45–49 age group, males had a significantly higher positivity rate (OR 0.89; 95% CI, 0.81–0.97; *p* = 0.011). Notably, males aged 45–49 years had the highest FIT positivity rate among all the male age groups (5.17%) (Figure 2).

### 3.2. Timing of Colonoscopy After Positive FIT

A total of 1973 individuals with positive FIT results underwent subsequent index colonoscopy. Among them, 78.2% underwent colonoscopy within 3 months following a positive FIT, whereas 81.9%, 83.6%, and 85.9% completed the procedure within 6-, 9-, and 12-month intervals, respectively. The risk of invasive cancer was not significantly reduced when the procedure was performed within 3 months (OR 0.63; 95% CI, 0.27–1.45; *p* = 0.272), 6 months (OR 0.49; 95% CI, 0.21–1.14; *p* = 0.098), 9 months (OR 0.43; 95% CI, 0.19–1.01; *p* = 0.052), or 12 months (OR 0.44; 95% CI, 0.184–1.058; *p* = 0.067).

### 3.3. Colonoscopic Findings

**Figure 3:**
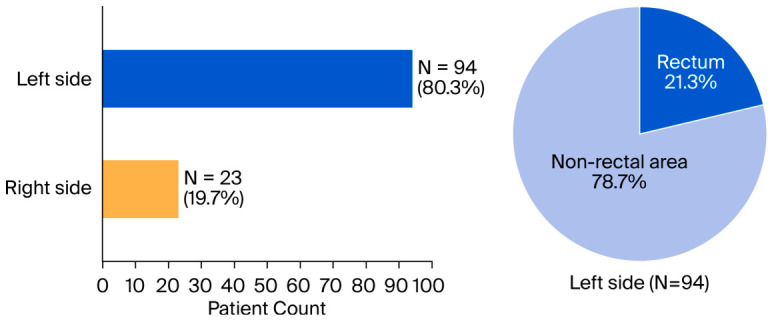
Distribution of advanced adenoma and advanced sessile serrated lesions.

According to the colonoscopic findings, 753 (38.2%) had colonic polyps, 569 (28.8%) had neoplastic colonic polyps, 117 (5.9%) had AA or ASSLs, and 26 (1.3%) had invasive cancer. A total of 143 individuals (7.2%) were diagnosed with ANs (96 males [67.1%] and 47 females [32.9%]). In 94 individuals (80.3%), the AA or ASSL was located on the left side of the colon, while for the remaining 19.7%, it was located on the right side. Among left-sided lesions, 20 (21.3%) were in the rectal area (Figure 3). Among these 117 individuals, 112 individuals had AA, of whom 97.3% had either tubular adenoma or tubulovillous adenoma, whereas five had ASSLs (Table 1). The optimal cutoff values for FIT in detecting ANs and invasive cancer were determined using the Youden index. Detailed diagnostic performance, including ROC curves and AUC values for different periods, is presented in the Supplementary Materials (Figure S1).

### 3.4. Age-Stratified Distribution of ANs

**Figure 4:**
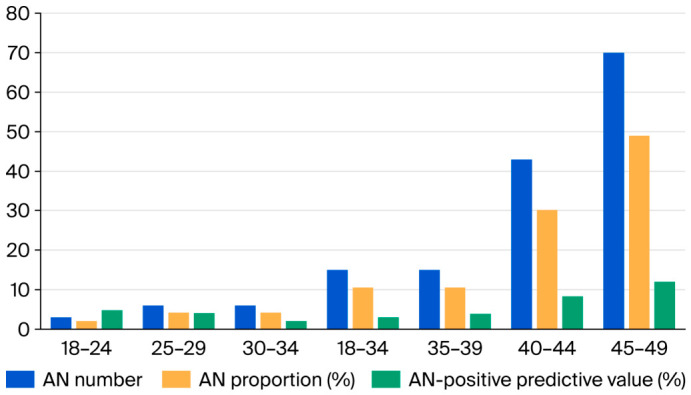
Age-related increase in advanced neoplasms (ANs).

Among all the ANs cases, three individuals (2.1%) were aged 18–24 years, six (4.2%) were aged 25–29 years, six (4.2%) were aged 30–34 years, 15 (10.5%) were aged 18–34 years, 15 (10.5%) were aged 35–39 years, 43 (30.1%) were aged 40–44 years, and 70 (49.0%) were aged 45–49 years. A total of 79.1% of AN cases occurred in individuals aged 40–49 years (Figure 4). When the prevalence of ANs was compared between adjacent age groups, a significantly greater prevalence was observed in the 45–49 age group than in the 40–44 age group (49.0% vs. 30.1%; OR 1.55; 95% CI, 1.04–2.32; *p* = 0.031). Similarly, AN prevalence was significantly higher in the 40–44 age group than in the 35–39 age group (30.1% vs. 10.5%; OR 2.20; 95% CI, 1.20–4.02; *p* = 0.011). After adjustment for total FIT-positive cases in each age group, the PPVs of ANs from lower to higher age groups were 4.8%, 4.1%, 2.1%, 3.9%, 8.3%, and 12.0%, respectively. When data for individuals aged 18–34 years were combined, the overall PPV of ANs in this younger cohort was 3.0% (Figure 4). These FIT results demonstrate a clear age-accelerated trend in AN prevalence and PPVs.

## 4. Discussion

In this single-center study of asymptomatic, average-risk adults, we observed a FIT positivity rate of 4.7%. Among those who underwent subsequent colonoscopy, ANs were predominantly detected in individuals aged 40–49 years, highlighting this age group as a potentially important population for CRC screening.

The biennial FIT-based Taiwan CRC screening program was launched in 2004 and has undergone several modifications, with the current program targeting the average population aged 45–74 years, and it continues to be effective in lowering CRC-specific mortality rates [10,11,12,18]. Compared to a one-time colonoscopy, the FIT has a less invasive nature and higher participation rates, while providing non-inferior 10-year CRC mortality risk, making it a more viable option for early-onset screening [19]. Globally, CRC screening programs for average-risk individuals have traditionally initiated screening at age 50 [13]. In response to the rising incidence of EOCRC, several countries have revised their screening recommendations. In the United States, the recommended starting age was officially lowered to 45 years in 2021, and Australia also lowered it to 45 years in 2024, while Japan has provided screening for adults starting at 40 years since 1992 [14,20,21]. The optimal age for CRC screening depends on the effectiveness and burden of screening capacities worldwide [22].

Nationwide data from Taiwan revealed that the overall FIT positivity rate ranged from 5.7% to 6.7% in those aged 50 years or older, with males exhibiting higher rates than females (7.3% vs. 4.5%). Among individuals aged 50–74 years, when stratified into five-year age intervals, the FIT positivity rates range from 4.5% to 7.7% and increase progressively with age [23]. These findings are consistent with previous global studies evaluating the feasibility of FIT, which reported positive rates ranging from 4.3% to 10.1% [24]. In our study, the overall FIT positivity rate was 4.7%. We observed a notable shift in the gender pattern across different age groups. In the younger population (ages 18–39), females had a significantly higher FIT positivity rate than males. However, this gender difference disappeared in the 40–44 age group. In the 45–49 age group, the trend completely reversed, with males showing a significantly higher rate, matching the typical pattern seen in older adults (≥50 years). This phenomenon could be partially explained by the interference of menstrual blood contamination in younger women, and this confounding effect likely diminishes as women enter the perimenopausal transition, typically beginning in the early to mid-40s.

In a previous single-center study from Taiwan, the prevalence of ANs among individuals aged 45–49 years was similar to that in the 50–54 age group, while later birth cohorts exhibited higher AN prevalence, as evidenced in both the 40–44 and 45–49 age groups [17]. In addition, data from the Taiwanese community-based national screening program showed that initiating FIT screening at ages 40–49 years resulted in greater and more sustained reductions in CRC incidence and mortality than starting at age 50, particularly given that the CRC incidence and mortality rates are higher among individuals aged 45–49 years than those aged 40–44 years [12]. In our study, a significant rise in AN prevalence was observed starting from the 40–44 age group, marking a distinct inflection point compared to the 18–39 age cohorts. While the prevalence remained lower than that of the 45–49 age group, the stable and comparable rates observed between the 18–34 and 35–39 age groups indicate age 40 as a critical threshold for initiating CRC screening.

In another study from Taiwan, a 60% FIT participation rate was associated with a marked 34% reduction in the incidence of advanced-stage CRC and a 40% reduction in the incidence of CRC-related death, with FIT screening being more effective in reducing distal than proximal neoplasms [18]. A nested case–control study conducted in the United States reported that undergoing at least one round of FIT screening was linked to a 33% reduction in CRC mortality, with an even greater reduction of 42% for cancers located in the distal colon and rectum [25]. Compared to left-sided tumors, the smaller benefit observed for right-sided tumors may be due to their rapid growth, the presence of fewer detectable lesions such as sessile serrated polyps, and the degradation of blood during longer transit from the proximal colon [18,25,26,27]. In our study, approximately 80% of AA or ASSLs were located on the left side of the colon, and approximately 20% of them were found in the rectum. This anatomical distribution aligns with the previous literature suggesting that FIT is particularly effective in detecting left-sided lesions, further supporting the utility of FIT-based screening in this younger population [18].

Among individuals with a positive FIT result, those who postponed their colonoscopy for more than 6 months had a higher rate of CRC detection than did those who completed the procedure within 6 months. The detection rate increased progressively with longer delays, reaching its peak when the interval exceeded 36 months [28]. In Taiwan, colonoscopy is also recommended within 6 months after a positive FIT result, as the incidence of CRC is relatively low among patients who receive a colonoscopy within this period, with approximately 5% of patients being diagnosed and only 1% being advanced-stage patients [29]. In our cohort, over 80% of FIT-positive individuals underwent subsequent colonoscopy within 6 months. Postponing the procedure for up to 12 months did not significantly increase the overall risk of invasive cancer. Our results suggest that younger FIT-positive individuals may possess a more manageable diagnostic buffer compared to the older populations described in the literature, potentially allowing for more flexibility in clinical follow-up in younger age groups.

With respect to EOCRC, somatic alterations and dysbiosis play critical roles owing to more westernized dietary patterns such as high consumption of processed meats and sugary drinks, increasing incidence of obesity and metabolic disorders, and common exposure to antibiotics. Compared to the overall CRC population, EOCRC patients have a greater prevalence of pathogenic germline variants, ranging from 16% to 25% [5,30]. Our study focused on age as the primary criterion for screening eligibility. This approach aligns with the current biennial FIT-based CRC screening program in Taiwan, which is primarily age-based to ensure population-wide accessibility and high participation.

This study has several limitations. First, the retrospective design may have introduced selection bias. Second, PPV was calculated only among FIT-positive individuals who completed a follow-up colonoscopy, which may have overestimated the predefined colorectal outcomes. Third, we did not include certain colorectal risk factors, which may influence the results. Even so, our findings provide important insight into the feasibility of applying the FIT screening in younger adults and its potential role in identifying ANs.

## 5. Conclusions

The overall FIT positivity rate in our young adult population was 4.7%, which was comparable to that reported in previous studies, even in populations over the age of 50. In response to the rising incidence of EOCRC, our findings highlight individuals aged 40–49 years as a key subgroup, as 79.1% of ANs were found in this age interval. While several countries have lowered the CRC screening initiation age from 50 to 45 years, our results provide additional population-based evidence to support consideration of screening strategies beginning at age 40.

## Figures and Tables

**Table 1:** Histopathological results of 117 patients with advanced adenoma or advanced sessile serrated lesions.

Results	Number (Proportion)
TA	35 (29.9)
TA + HGD	1 (0.9)
TVA	54 (46.2)
TVA + HGD	13 (11.1)
TVA + Intramucosal adenocarcinoma	6 (5.1)
HGD + Intramucosal adenocarcinoma	1 (0.9)
Intramucosal adenocarcinoma	2 (1.7)
TSA	3 (2.6)
SSL	2 (1.7)

HGD, high-grade dysplasia; SSL, sessile serrated lesion; TA, tubular adenoma; TSA, traditional serrated adenoma; TVA, tubulovillous adenoma.

## Data Availability

The data that support the findings of this study are available from the corresponding author upon reasonable request.

## Supplementary Materials

The following supporting information can be downloaded at https://www.mdpi.com/article/10.3390/jcm15114293/s1. Figure S1: Comparison of the diagnostic performance of fecal immunochemical test (FIT) for advanced neoplasms (ANs) and invasive cancer across two study periods (2011–2021 vs. 2021–2025); Table S1: FIT positivity rate (%) in each age group.

## Abbreviations

The following abbreviations are used in this manuscript:

AA	Advanced adenoma
ANs	Advanced neoplasms
ASSL	Advanced sessile serrated lesion
AUC	Area under the curve
CIs	confidence intervals
CRC	Colorectal cancer
EOCRC	Early-onset colorectal cancer
FIT	Fecal immunochemical test
HGD	High-grade dysplasia
ORs	Odds ratios
PPV	Positive predictive value
ROC	Receiver operating characteristic
SSL	Sessile serrated lesion
TA	Tubular adenoma
TSA	Traditional serrated adenoma
TVA	Tubulovillous adenoma
