# Supplementary material for: Diagnostic Yield of Fecal Immunochemical Test for Advanced Colorectal Neoplasms in Adults Under 50: A Single-Center Cohort in Taiwan
Source: J Clin Med. 2026 Jun 2;15(11):4293. doi: 10.3390/jcm15114293 (PMC13257418; doi:10.3390/jcm15114293)
Supplement: Supplementary file 1 [file jcm-15-04293-s001.zip › jcm-4298401-supplementary.pdf]

Table S1. FIT positivity rate (%) in each age group.

Age Group	Both Gender	Female/Male	OR 95% CI	*p* Value
18–24	6.03	7.45/4.75	1.61 (1.29–2.01)	<0.001
25–29	4.91	5.59/4.37	1.29 (1.16–1.45)	<0.001
30–34	4.40	4.95/3.99	1.26 (1.14–1.39)	<0.001
35–39	4.52	4.81/4.31	1.12 (1.02–1.23)	0.015
40–44	4.48	4.62/4.37	1.06 (0.97–1.15)	0.194
45–49	4.95	4.62/5.17	0.89 (0.81–0.97)	0.011
18–49	4.70	4.94/4.47	1.11 (1.07–1.16)	<0.001

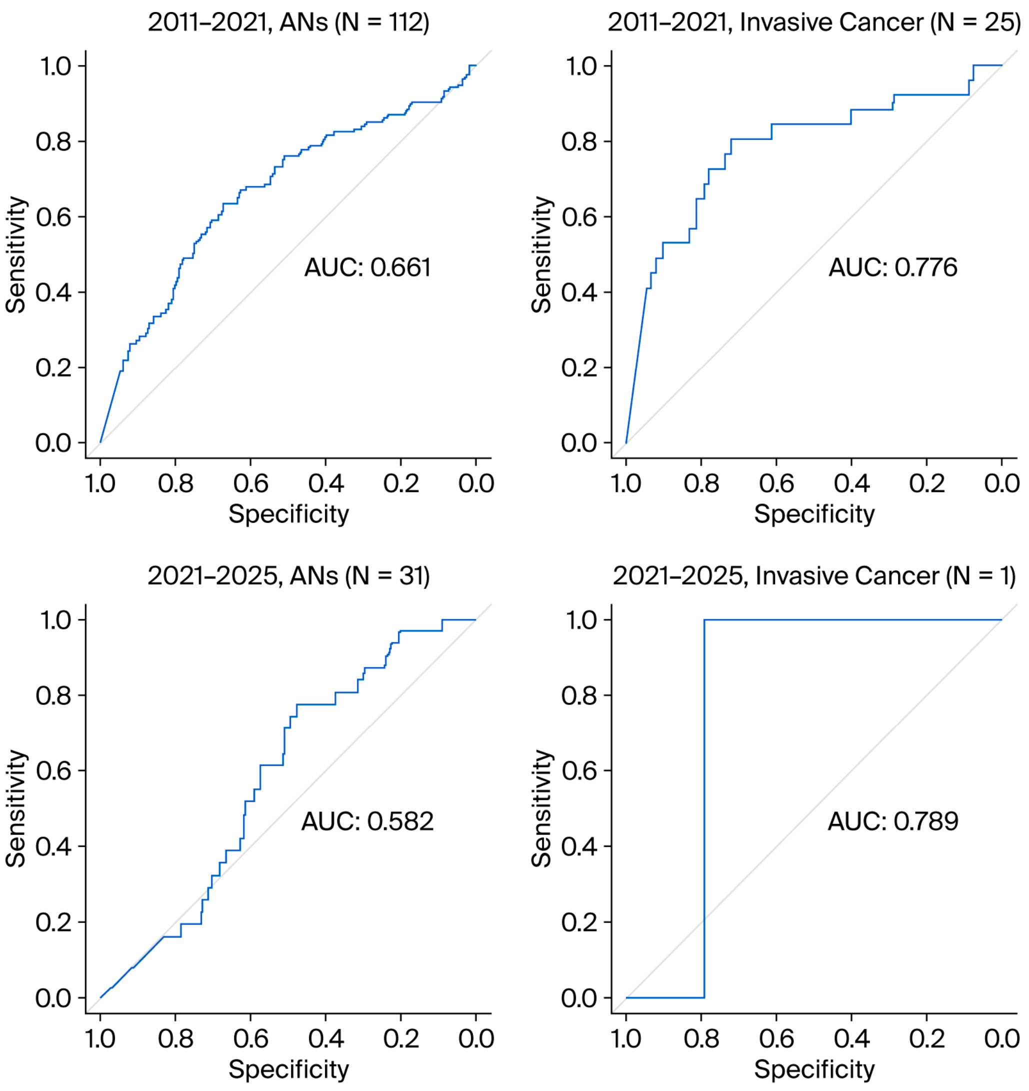

Figure S1. Comparison of the diagnostic performance of FIT for advanced neoplasms (ANs) and invasive cancer across two study periods (2011–2021 vs. 2021–2025).

From 2011 to 2021, the diagnostic performance of the fecal immunochemical test (FIT) for advanced neoplasms (ANs) (N=112) yielded an area under the receiver operating characteristic curve (AUC) of 0.661. Using the Youden index to determine the optimal cutoff, the threshold was identified at 72.55 μg hemoglobin (Hb)/g feces, providing a sensitivity

 https://doi.org/10.3390/jcm15114293

of 63.1% and a specificity of 67.1%. For the detection of invasive cancer (N=25) during the same period, the FIT demonstrated a superior diagnostic accuracy with an AUC of 0.776. The optimal threshold determined by the Youden index was 98.15 μg Hb/g feces, resulting in a higher sensitivity of 80.0% and a specificity of 71.8%. For the period of 2021–2025, the FIT demonstrated a limited diagnostic accuracy for ANs (N=31), with an AUC of 0.582. Using the Youden index, the optimal threshold was determined to be 230.5 μg Hb/g feces, yielding a sensitivity of 77.4% and a specificity of 47.5%. In contrast, the performance of FIT in detecting invasive cancer (N=1) during the same period showed an AUC of 0.789. At the optimal threshold of 703.5 μg Hb/g feces, the test achieved a sensitivity of 100.0% and a specificity of 78.9%.

https://doi.org/10.3390/jcm15114293
